# Towards accurate artificial intelligence models for strain-level phage–host prediction

**DOI:** 10.1093/bib/bbag085

**Published:** 2026-02-26

**Authors:** Chris J Malajczuk, Andrew Vaitekenas, Joshua J Iszatt, Stephen M Stick, Anthony Kicic, Yuliya V Karpievitch

**Affiliations:** Wal-yan Respiratory Research Centre, The Kids Research Institute Australia, Western Australia, Perth, 6009, Australia; UWA Centre for Child Health Research, University of Western Australia, Western Australia, Perth, 6009, Australia; Wal-yan Respiratory Research Centre, The Kids Research Institute Australia, Western Australia, Perth, 6009, Australia; UWA Centre for Child Health Research, University of Western Australia, Western Australia, Perth, 6009, Australia; Wal-yan Respiratory Research Centre, The Kids Research Institute Australia, Western Australia, Perth, 6009, Australia; UWA Centre for Child Health Research, University of Western Australia, Western Australia, Perth, 6009, Australia; Department of Respiratory and Sleep Medicine, Perth Children’s Hospital, Western Australia, Perth, 6009, Australia; Wal-yan Respiratory Research Centre, The Kids Research Institute Australia, Western Australia, Perth, 6009, Australia; UWA Centre for Child Health Research, University of Western Australia, Western Australia, Perth, 6009, Australia; School of Population Health, Curtin University, Western Australia, Perth, 6102, Australia; Wal-yan Respiratory Research Centre, The Kids Research Institute Australia, Western Australia, Perth, 6009, Australia; School of Population Health, Curtin University, Western Australia, Perth, 6102, Australia; School of Biomedical Sciences, University of Western Australia, Western Australia, Perth, 6009, Australia

**Keywords:** phage–host interactions, bacteriophage, strain-level prediction, artificial intelligence, predictive modelling, phage therapy

## Abstract

Strain-level prediction of phage–host interactions (PHIs) is essential for developing targeted phage therapies. Traditional empirical and homology-based methods often lack the resolution and scalability needed for precision applications. Recently, a new generation of artificial intelligence-driven models has emerged leveraging genomic information to infer PHIs at strain-level resolution. Here, we review recent advances in strain-level PHI prediction, spanning biologically grounded feature-based models, hybrid representation-learning frameworks, phylogeny-agnostic machine learning approaches, and end-to-end deep learning architectures. We examine how these modelling strategies navigate shared structural constraints arising from sparse and imbalanced outcome data, assay-dependent labels, infection complexity, and limited generalization. We further analyse how evaluation design, negative definition, and train-test splitting strategies shape apparent strain-level performance, and why inappropriate benchmarking can inflate claims of biological resolution. Framing these issues in the context of clinical phage therapy, we examine how current strain-level PHI prediction frameworks perform under the biological, experimental, and data constraints characteristic of real-world therapeutic settings. Finally, we outline pragmatic pathways toward more robust, interpretable, and clinically translatable PHI prediction systems.

## Introduction

Phage-based technologies, including phage therapy, are emerging as precision tools to combat the growing threat of antibiotic-resistant bacteria [1–3]. Central to their success is the accurate and rapid prediction of phage–host interactions (PHIs), especially at the strain (intraspecies) level, where bacterial susceptibility can hinge on subtle genomic and structural variation [4, 5]. Many phages display narrow host ranges, sometimes infecting only a few strains within a species [6], making the identification of therapeutically effective phages a non-trivial and increasingly data-intensive task. This has driven growing interest in scalable, fine-grained PHI prediction methods to streamline phage candidate selection.

Two biological concepts underpin PHI prediction: host range and infection outcome. Host range describes the set of bacterial strains a phage can recognize and infect, shaped by receptor compatibility, bacterial defence systems, and phage-encoded countermeasures. Infection outcomes span failed adsorption, abortive infection, lysogeny, or productive lytic replication. For therapeutic applications, productive lytic infections are typically prioritized, as they result in efficient bacterial clearance while avoiding horizontal gene transfer and other risks associated with lysogeny [7].

**Figure 1:**
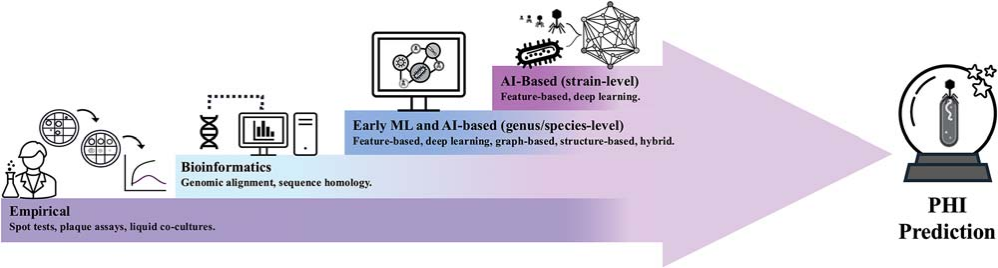
Evolution of methodologies for predicting PHIs. Step 1: Traditional experimental laboratory assays are foundational for all PHI predictions but are limited in their reproducibility and scalability prompting the development of early computational approaches. Step 2: Alignment-based bioinformatics methods partially addressed scalability but are limited by dependency on known interactions and genomic annotations. Steps 3 and 4: Modern ML- and AI-based models have emerged as sophisticated alternatives, capable of scalable predictions, generalizable beyond known interactions, and uniquely able to achieve genus-, species-, and strain-level specificity; yet still present distinct challenges.

Experimentally, PHIs are assessed using microbiological assays such as spot tests, plaque assays, and liquid co-culture growth experiments (time-kill or ‘kill curve’ assays). While foundational, these approaches are labor-intensive and often inconsistent across studies due to variation in host panels, growth conditions, and outcome interpretation [8, 9]. Such variability complicates data integration and limits the standardization of host-range datasets, yet these empirical measurements remain indispensable and form the basis of all PHI prediction frameworks (Fig. 1; bottom step).

Early computational approaches to PHI prediction relied primarily on bioinformatics-based similarity methods, including sequence alignment and CRISPR spacer matching, to infer interactions by homology to previously annotated phage–host pairs [10, 11] (Fig. 1; second step). Tools such as PhARIS [12], which compare phage receptor-binding proteins (RBPs) against curated reference sets, can achieve high accuracy in well-characterized systems. However, these approaches struggle to generalize to novel phages or under-explored bacterial strains, particularly where receptor profiles or defence systems are poorly annotated. Small sequence changes in RBPs [13] or genes involved in overcoming host defences [14] can substantially alter infection outcomes, limiting the robustness of purely homology-based inference.

To address these limitations, a growing body of artificial intelligence-based models has emerged to improve the scalability, flexibility, and generalizability of PHI prediction (Fig. 1; top two steps). Throughout this review, we use the term ‘artificial intelligence’ (AI) broadly to encompass data-driven approaches including classical machine learning (ML), ensemble methods, graph-based models, and deep learning (DL). Where relevant, we refer explicitly to specific architectural paradigms to avoid conflating general AI-driven inference with particular modelling choices.

Contemporary AI-based PHI predictors integrate diverse biological signals, including nucleotide composition, protein domains, and ecological or network-derived features, and span a wide architectural spectrum from feature-based ML to DL, graph neural networks, and hybrid frameworks. While these methods overcome some limitations of homology-based tools by learning latent patterns directly from data, most have been developed for genus- or species-level prediction rather than strain-level resolution [8, 15]. Recent reviews by Nie *et al.* [15] and Howell *et al.* [8] provide comprehensive overviews of these first- and second-generation models, detailing their architectures, input features, and taxonomic scope.

Tools such as vHULK [16], RaFAH [17], and PHIAF [18], along with alignment-free feature-based approaches such as PredPHI [19] and PB-LKS [20], have introduced ensemble learning, k-mer composition, protein clustering, and synthetic data augmentation, achieving high performance in genus-level and metagenomic settings. Other tools including DeepHost [21], PHISGAE [22], DSPHI [23], PHPGAT [24], and GE-PHI [25] illustrate emerging methodological trends such as attention-based learning, graph-based inference, and multimodal integration. However, the utility of these models for clinical applications requiring fine-grained specificity and biological interpretability remains limited.

AI-based models explicitly developed for strain-level prediction are typically designed for specific pathogens or phage systems and often require iterative updating to accommodate the rapid evolution of phage–host landscapes (Fig. 1; top step). It is therefore important to distinguish strain-level PHI prediction from the application of broader taxonomic-level models to strain-resolved datasets. Several predictors primarily developed for genus- or species-level tasks, including PredPHI [19], PHIAF [18], PB-LKS [20], and PHPRBP [26] have been evaluated on strain-resolved PHI matrices or rank-stratified benchmarks and can recover predictive signal under certain conditions [20, 26, 27]. However, these models were not explicitly designed to resolve fine-scale host specificity between closely related bacterial isolates, and observed performance may reflect implicit exploitation of phylogenetic structure, taxonomic constraints, or dataset composition, rather than genuine discrimination of strain-specific genomic compatibility. Throughout this review, we therefore reserve the term ‘strain-level PHI prediction’ for models explicitly developed and evaluated to resolve strain-specific compatibility using genomic or sequence-derived information.

**Figure 2:**
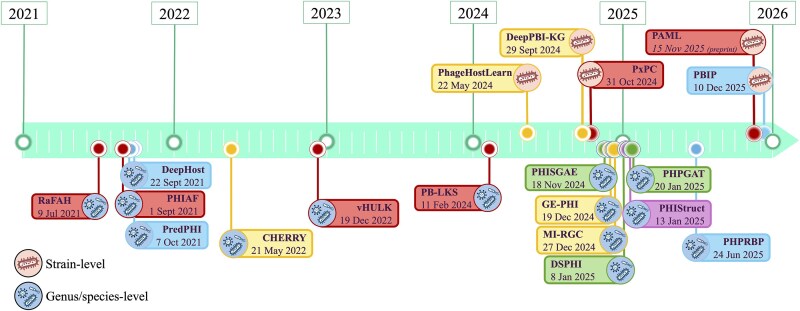
Timeline of the notable AI-based PHI prediction models from the last 5 years, with strain-level models shown above the timeline and species/genus-level models below. Models are coloured according to model architecture with red flags for feature-based, blue flags for DL, green flags for graph neural-network, yellow flags for hybrid, and purple flags indicating a structure-based approach.

The emergence of AI-based strain-level PHI prediction frameworks across the past 2 years coincides with continued progress in species- and genus-level models (Fig. 2), reflecting not a shift away from broader taxonomic inference, but a maturation and diversification of the field. As data availability, computational capacity, and representation learning techniques have advanced, PHI prediction has begun to branch toward increasingly specialized and application-driven objectives, including high-resolution tasks such as rational phage cocktail design for therapy [6]. This diversification underscores the need for careful assessment of how modelling assumptions, data structures, and evaluation strategies differ across resolution scales.

Accordingly, this review focuses specifically on AI-based models developed for and benchmarked against strain-level PHI prediction tasks. Our aims are to (i) critically evaluate the methodological design, predictive scope, and biological assumptions of current strain-level PHI models, and (ii) identify cross-cutting challenges and strategic priorities for advancing their precision, interpretability, and clinical applicability. By clarifying the emerging methodological landscape and its associated trade-offs, we seek to guide the development of strain-level PHI prediction tools that are not only accurate, but also biologically grounded and practically deployable.

## Biological and methodological challenges in phage–host interaction prediction

**Figure 3:**
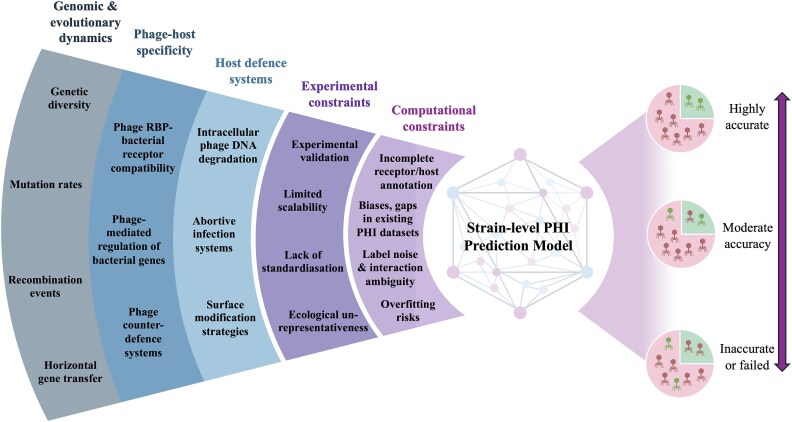
Biological and methodological challenges in predicting PHIs. Accurately predicting PHIs is complicated by multiple interdependent upstream biological and methodological factors that feed into the strain-level PHI prediction model to ultimately inform predictive accuracy.

Accurate prediction of PHIs at strain-level resolution is constrained by biological complexity, experimental limitations, and structural deficiencies in available datasets. These include sparse strain-resolved PHI matrices, inconsistent metadata, taxonomic bias toward model organisms, and heterogeneity in interaction labelling. Many of these challenges are not merely amplified but fundamentally altered at strain resolution, where subtle biological differences exert disproportionate influence on infection outcome. Together, these factors have impeded the development of generalizable and scalable PHI prediction tools (Fig. 3). This section briefly outlines the key biological and methodological barriers underlying this limitation.

### Complexities of phage biology and host specificity

Phages exhibit extensive genetic diversity, rapid evolutionary dynamics, and high ecological adaptability [28, 29]. Host specificity is typically initiated through binding between phage-encoded RBPs and specific host cell surface receptors [6, 30, 31], yet even minor variation in receptor structure, expression, or regulation can generate pronounced strain-level differences in susceptibility [32, 33]. At this resolution, micro-variation rather than overall genomic similarity often determines infection outcome [34–39].

Importantly, successful adsorption does not guarantee productive infection. Following entry, phages must overcome multiple intracellular barriers, including restriction-modification systems, CRISPR-Cas immunity, abortive infection pathways, and blocks to transcription, translation, or assembly [40]. Phages may counter these defences through dedicated anti-defence systems [30, 41] or broader regulatory manipulation, such as sigma factor hijacking and anti-repressor activity [42, 43]. These interactions are highly context-dependent [44] and shaped by evolutionary trade-offs. For example, amplification of the anti-defence gene *tifA* in T4 phages has been shown to coincide with compensatory loss of accessory genes important for infecting alternative hosts, thereby constraining host range [34].

Such dynamics reflect the evolutionary plasticity of phage genomes, which evolve rapidly via mutation, recombination, and horizontal gene transfer [45]. Even small genomic changes, including point mutations [35–37] or domain swaps [38, 39], can substantially alter infection capacity, limiting the reliability of predictions based solely on global sequence similarity.

### Experimental constraints and dataset limitations

Laboratory assays remain the foundation of PHI determination, with spot tests, plaque assays, and liquid co-culture methods providing complementary perspectives on infection outcomes [46, 47]. Semi-quantitative measures such as efficiency of plating [48], or virulence and centroid indices derived from growth curves [49, 50], offer additional resolution, but each assay carries intrinsic limitations affecting reproducibility, interpretability, and scalability.

Assay outcomes are highly sensitive to experimental conditions, including growth medium, host physiological state, bacterial density, and incubation parameters [51]. Minor protocol variations can substantially alter observed infection dynamics [52, 53], complicating cross-study comparison and introducing hidden heterogeneity into PHI datasets.

Moreover, many commonly used assays prioritize visible lysis rather than mechanistic resolution of infection outcome. Spot tests, e.g. can overestimate virulence by conflating productive lysis with abortive infection or lysis from without [52], while apparent non-infective outcomes may reflect incomplete adsorption, suppressed replication, or alternative infection modes [54–57]. Without follow-up assays such as adsorption measurements, time-resolved growth monitoring, or sequencing, these biologically distinct states remain collapsed into coarse outcome labels, contributing to label noise and reduced interpretability in downstream models.

Assay accessibility further shapes dataset composition. Interactions involving slow-growing, fastidious, or difficult-to-culture strains are systematically underrepresented [58, 59], biasing PHI datasets towards well-characterized laboratory strains [60–62]. In clinical contexts, however, many priority pathogens fall precisely into these underrepresented categories [63–65], limiting the relevance of existing datasets for translational prediction tasks.

### Data quality and taxonomic coverage

The performance and generalizability of PHI prediction models depends critically on the quality and representativeness of training data. Yet most available datasets exhibit limited strain-level resolution, inconsistent annotation practices, and uneven taxonomic coverage. Public resources such as the Viral Host Range Database [61] and Virus–Host DB [62] remain heavily skewed toward model genera such as *Escherichia* and *Klebsiella*, reflecting historical research focus rather than clinical need.

A major limitation is the absence of standardized frameworks for defining and reporting PHI outcomes. Interaction results are frequently reduced to binary or semi-quantitative labels without clear distinction between productive and non-productive infections. While such ambiguity may be tolerated at broader taxonomic scales [66], it becomes a dominant failure mode at strain resolution, where small phenotypic differences carry substantial predictive weight.

Additional data-quality challenges include missing or inconsistent metadata, non-standardized isolate identifiers, and incomplete reporting of experimental parameters such as multiplicity of infection, incubation time, or phage titre. These deficiencies hinder dataset integration, benchmarking, and reuse, further constraining the development of robust and generalizable strain-level PHI prediction models.

## Artificial intelligence-based models for strain-level phage–host interaction prediction

The challenges outlined in Section 1 impose stringent and often competing demands on strain-level PHI prediction models. Effective frameworks must operate under sparse and noisy supervision, accommodate assay-dependent outcome labels, and resolve fine-scale host specificity without overfitting to lineage structure or experimental artefacts. In response, a small but rapidly evolving set of AI-based models has emerged, each reflecting distinct assumptions about how biological knowledge should be encoded, how uncertainty should be handled, and how predictive power should be balanced against interpretability and data availability.

Existing strain-level predictors span a continuum of architectural and representational strategies. These include the Phage-by-Phage Classifier (PxPC) [6], a supervised ML model based on curated genomic features and semi-quantitative lytic outcomes; PhageHostLearn [67], which integrates phage RBP and bacterial capsular protein sequence embeddings in a pairwise learning framework; DeepPBI-KG [68], which applies deep neural networks to engineered, system-specific genomic features; a phylogeny-agnostic ML framework (PAML) that leverages large-scale, feature-agnostic genomic representations across diverse taxa [69]; and the Phage–Bacterium Interaction Predictor (PBIP) [27], an end-to-end DL model that learns strain-level compatibility directly from pretrained protein sequence embeddings. A comparative summary of these models is provided in Table 1.

**Table 1:** Summary table of AI-based strain-level PHI prediction models.

**Model**	**Host taxonomy**	**PHI data**					**Model input**			**Predictor/** **classifier**	**Evaluation/** **train-test strategy**	**Evaluation metrics**	**Primary application/design intent**
		**N. Phages,**	**N. Strains**	**Total PHIs (positive PHI %)**	**Assay type**	**Class imbalance handling**	**Sequence type**	**Features / embeddings**	**Pair representation**				
PxPC Gaborieau *et al*. [6]	*Escherichia coli*	96	403	36 688 (20.6%)	Multi-concentration plaque assay with semi-quantitative MLC scoring; binarized to lytic (MLC > 0) and non-lytic (MLC = 0).	Per-phage class weighting during training; evaluation includes PR-based metrics; model selection by AUROC.	Genome	Curated, biologically motivated binary/categorical features (no learned embeddings).	Phage-specific modelling: one model per phage; each predicts infectivity from the host feature vector (pair handled implicitly by per-phage framing).	Random Forest classifier per phage; outputs probability of lytic infection.	Phage-wise cross-validation within fixed host–phage system.	AUROC (model selection), plus precision, recall, F1, average precision (AUPRC).	Rational phage cocktail design and prioritization for *E. coli*
PhageHostLearn Boeckaerts *et al.* [67]	*Klebsiella spp.*	105	200	10 006 (3.3%)	Spot test screening with planktonic killing assay confirmation (growth inhibition); binarized to productive / non-productive.	Not explicitly described as resampling/weighting; trained/evaluated on sparse positives with ranking-style evaluation.	Proteome	ESM-2 embeddings per protein; mean pooling to phage- and host-level vectors.	Concatenate mean-pooled phage RBP vector with mean-pooled bacterial capsule vector (fixed-length).	XGBoost classifier on concatenated embeddings.	Stratified PHI-pair cross-validation within system.	ROC AUC and hit ratio@k (retrieval/ranking).	Pairwise host-range prediction to support phage prioritization.
DeepPBI-KG Wei *et al.* [68]	*Klebsiella pneumonia*	110	113	12 430 (25.5%)	Spot test screening; binarized to productive / non-productive.	Explicit negative sampling/balancing; retraining with controlled class splits.	Genome & proteome	Engineered sequence-derived features (protein + DNA descriptors).	Concatenation of engineered phage and host key-gene features into one vector.	Deep neural network (multi-layer fully connected).	Held-out validation set of unseen *K. pneumonia* phage–strain pairs.	AUROC, AUPRC.	General PHI prediction with retraining for intraspecies host-range analysis.
PAML Arkin *et al.* [69]	*Escherichia coli*	94	402	37 788 (20.7%)	Same multi-concentration plaque assay with semi-quantitative MLC scoring as PxPC but excluding non-infections phages and no-infection strains; binarized to lytic (MLC > 0) and non-lytic (MLC = 0).	Phage-specific inverse-frequency class weights; emphasizes imbalance-aware evaluation (MCC/PR).	Genome	Engineered genome features: protein family presence/absence + k-mer composition, with feature filtering/reduction.	Concatenation of independent phage and bacterial genome feature vectors into a single fixed-length feature vector per interaction.	CatBoost gradient boosting reported as best-performing.	Conservative PHI-pair splitting with imbalance-aware evaluation.	Emphasizes MCC; also reports AUROC/PR-style evaluation.	Phylogeny-agnostic host-range prediction for broad screening and downstream cocktail design
	*Klebsiella pneumonia*	59	62	3658 (4.9%)	Replicated spot tests with dilution-based confirmation of productive infection; binarized to lytic/non-lytic.								
	*Mixed Klebsiella spp.*	46	138	6348 (2.4%)	Binarized spot tests.								
	*Pseudomonas aeruginosa*	19	23	437 (36.2%)	Quantitative efficiency of plating; binarized to lytic/non-lytic.								
	*Mixed Vibronaceae*	248	256	63 488 (2.1%)	Consensus spot testing validated by planktonic killing assays with phage progeny confirmation; binarized to lytic/non-lytic.								
**PBIP** Ma *et al.* [27]	*Klebsiella pneumonia*	104	120	12 480 (22.8%)	Spot tests with associates ‘transmissivity’ score 0–3, averaged across replicates and thresholded to binary.	Explicitly evaluates SMOTE during benchmarking; balanced test set; discusses imbalance sensitivity across models.	Proteome	UniRep embeddings per protein; mean pooling across all proteins to fixed phage and host vectors.	Concatenate pooled vectors.	DL architecture: CNN + Bi-GRU + attention.	Random PHI-pair splits with data augmentation; no external validation.	AUROC, AUPRC, ACC, precision, recall, F1; plus benchmarking comparisons.	High-resolution strain-level infectivity prediction for clinical phage selection.

### Phage-by-phage classifier, a feature-based model

Feature-based AI approaches to PHI prediction encode prior biological knowledge into supervised learning frameworks using curated genomic descriptors. At strain-level resolution, their success depends on the specificity and relevance of selected features, annotation quality, and the reliability of interaction labels. PxPC, introduced by Gaborieau *et al.* [6], provides a clear example of how biologically grounded feature selection can support accurate strain-level PHI prediction when paired with systematically generated experimental data.

PxPC was developed for virulent *Escherichia* phages*,* leveraging a system with well-defined adsorption phenotypes and systematically curated host-range data [6]. Rather than training a single global model, PxPC adopts a phage-specific strategy, training independent binary classifiers for each of 96 phages. Each model predicts susceptibility of *Escherichia coli* strains to a given phage, explicitly reflecting the narrow host ranges typical of these phages [6] and allowing phage-specific interaction patterns to be learned without dilution in pooled models.

Computationally, PxPC models phage-host compatibility implicitly through host-side genomic features. Bacterial genomes are encoded as fixed-length vectors of binary presence-absence indicators representing surface-associated adsorption determinants, including O-antigen serotypes, lipopolysaccharide (LPS) outer-core types, and ABC-transporter-dependent capsule (K-antigen) loci. These features were derived through systematic genomic annotation pipelines [6, 70–73]. Candidate defence systems were also identified via DefenseFinder [74] and evaluated for predictive signal, however these features were excluded from the final feature set due to their limited predictive value in this *Escherichia* system [6].

Encoding adsorption determinants as binary features affords interpretability and computational simplicity but treats receptor identity as categorical, without capturing fine-scale sequence or structural variation known to influence RBP-receptor compatibility within receptor classes [75–77]. PxPC therefore resolves coarse-grained adsorption compatibility rather than molecular interaction detail.

A major strength of PxPC lies in its training data. The authors generated a dense, internally consistent interaction matrix by systematically screening all pairwise combinations between 403 *E. coli* isolates and 96 phages using plaque assays, yielding 38 688 validated PHIs (20.6% considered lytic interactions) [6]. Interactions were scored using a minimum lytic concentration (MLC) metric and subsequently binarized for model training, a pragmatic choice that stabilized learning under extreme class imbalance and aligned with downstream goals such as phage cocktail design [6].

Within this system, PxPC achieved high predictive performance (AUROC ≈ 0.85–0.86) and has since served as a benchmark dataset for subsequent strain-level modelling efforts, including PAML [69]. Overall, PxPC illustrates how mechanistic priors and high-quality outcome data can enable interpretable strain-level PHI prediction within well-characterized systems, while also foregrounding scalability and transferability trade-offs revisited throughout Section 3.

### PhageHostLearn, a deep learning-augmented feature-based model

DL offers a route to higher-resolution PHI prediction by learning distributed representations of biological sequences that extend beyond manually curated features. In principle, pretrained protein language models (pLMs) can encode evolutionary and contextual constraints without explicit annotation or alignment [78]. In practice, however, the scarcity of large, consistently labelled strain-level PHI datasets and the combinatorial complexity of PHIs have limited the applicability of fully end-to-end DL models. As a result, recent strain-level approaches have favoured hybrid strategies that integrate pretrained sequence embeddings within biologically informed supervised learning frameworks [67, 78].

PhageHostLearn [67] exemplifies this design philosophy. Rather than encoding adsorption determinants as binary features, the model represents key adsorption-related proteins using pretrained pLM embeddings while retaining a conventional ML classifier. As with PxPC, PhageHostLearn is grounded in a clear biological assumption: that productive infection in *Klebsiella* is strongly influenced by interactions between phage RBPs and the bacterial capsule. Host-side representation is therefore restricted to K-locus (capsular) proteins, reflecting the central role of capsule diversity in *Klebsiella* phage susceptibility [79, 80].

Operationally, genes in each phage genome are predicted using PHANOTATE [81] followed by domain-based RBP detection to identify putative RBPs among translated proteins (filtered to 200–1500 aa) [82]. In parallel, bacterial capsule K-locus proteins are identified using Kaptive [83, 84]. Protein sequences from both sides are embedded using the ESM-2 transformer-based pLM [85]. For each phage and host, embeddings are mean-pooled across all detected RBPs or capsule proteins, concatenated to form a phage–host pair representation, and classified using a binary XGBoost model [86].

This architecture balances interpretability and representational richness. Compared with the binary adsorption features used in PxPC, embedding-based representations relax assumptions about feature identity and allow graded similarity between related protein variants to influence predictions. However, aggregation through pooling necessarily summarizes adsorption machinery at a coarse level, collapsing heterogeneity among individual RBPs or capsule proteins. As a result, PhageHostLearn models average adsorption compatibility rather than resolving fine-grained protein-specific determinants, a limitation revisited in Section 3.2.

PhageHostLearn was trained on 10 006 pairwise spot–test interactions between 105 *Klebsiella* phages and 200 clinical *Klebsiella* strains, yielding 333 (3.33%) positive interactions [67, 79]. In cross-validation, PhageHostLearn achieved an AUROC of 0.818 [67]. Prospective evaluation on 28 multidrug-resistant *Klebsiella pneumoniae* clinical isolates yielded comparable performance (AUROC 0.793), with at least one experimentally confirmed interaction identified for 16 isolates following targeted validation of top-ranked predictions [67].

Validation analyses highlighted key limitations. Several predicted interactions were only observed at high phage titres, consistent with incomplete or non-productive infection states, and no consistent associations were detected between intracellular defence systems and infection outcomes. These findings underscore that adsorption-focused representations alone may not fully resolve post-adsorption barriers. Accordingly, the authors proposed a layered modelling strategy in which PhageHostLearn serves as an adsorption-centric first stage, to be complemented by predictors targeting intracellular defence mechanisms [67].

Overall, PhageHostLearn occupies an intermediate position between strictly feature-based models such as PxPC and fully representation-driven DL approaches. Its performance demonstrates the value of integrating learned sequence representations with mechanistic priors under current data constraints, while also highlighting the need for broader representations to capture downstream and condition-dependent infection determinants.

### Feature-agnostic and data-driven machine learning

Beyond biologically constrained, adsorption-centric models, recent work has explored whether strain-level PHI prediction can be achieved by relaxing explicit mechanistic assumptions about infection determinants. Two representative approaches occupying this intermediate design space are DeepPBI-KG [68] and the PAML framework [69]. While both move beyond narrowly defined adsorption features, they reflect contrasting philosophies regarding how biological knowledge, interaction uncertainty, and dataset scale should be incorporated into strain-level prediction.

DeepPBI-KG [68] adopts a feature-engineered, data-driven strategy in which strain-level PHIs are inferred from curated genomic descriptors rather than direct modelling of specific infection mechanisms. Rather than operating directly on raw genome sequences, the framework constructs structured representations of phage and host genomes by first identifying subsets of ‘key genes’ associated with PHIs and then summarizing sequence-derived properties of those genes prior to classification. In the authors’ workflow, phage and bacterial genomes are annotated using Prokka [87] to identify coding sequences, and translated proteins are additionally screened against conserved domain databases to support biological interpretation and validation of candidate RBPs.

Feature construction proceeds in two stages. First, auxiliary gene-count vectors are built for phages and hosts, encoding the presence and frequency of gene types across genomes. These concatenated phage-host vectors are subjected to ensemble-based feature-importance ranking using random forests, and reduced gene sets are retained based on cumulative importance thresholds. Second, for coding sequences corresponding to these selected genes, a comprehensive panel of DNA- and protein-level descriptors is computed and aggregated using summary statistics to produce fixed-length genome representations. Phage and host feature vectors are then concatenated to form pairwise inputs for classification using a multi-layer neural network.

This design substantially expands the representational scope beyond the small set of curated adsorption features used in PxPC [6], allowing intracellular and non-adsorptive determinants to contribute indirectly through distributed genomic signal. However, unlike PhageHostLearn [67], representation learning is not performed end-to-end from sequence. Instead, predictive performance remains tightly coupled with annotation quality, feature-engineering choices, and system-specific gene selection, limiting transferability beyond the target host system.

DeepPBI-KG also adopts an aggressive strategy to address outcome uncertainty and class imbalance. Negative interactions are inferred from untested phage–host pairs, and balanced training sets are constructed via clustering-based subsampling of negatives. While these steps stabilize optimization and yield strong apparent performance within the *Klebsiella* system, they entangle predictive success with dataset construction and sampling strategy, complicating interpretation of reported metrics and limiting evidence for broader generalizability. Within its target system, DeepPBI-KG outperformed several retrained species-level baselines, including PredPHI and PHIAF [68], but remains best understood as a locally optimized, system-specific solution.

In contrast, PAML [69] was explicitly designed to test whether strain-level PHI prediction could be achieved without reliance on curated receptor annotations, host taxonomy, or predefined infection mechanisms. Rather than optimizing for a single bacterial system, PAML aggregates heterogeneous PHI datasets spanning multiple genera and experimental contexts while retaining strain-resolved interaction labels.

Genomic representations in PAML are derived directly from whole-genome sequences using annotation-light feature spaces. Specifically, phage and bacterial genomes are represented as protein family presence-absence profiles generated by clustering predicted coding sequences with MMSeqs2 [88] across the full dataset, producing a pangenome-like binary feature matrix in which each dimension corresponds to a protein family rather than a predefined functional category. In parallel, amino-acid k-mer composition features are computed directly from translated protein sequences, enabling sequence-level variation to contribute to prediction without requiring homology-based annotation. Importantly, neither representation assumes prior knowledge of receptors, RBPs, or defence systems.

Given the resulting high-dimensional feature space and extreme outcome sparsity, PAML places substantial emphasis on feature filtering, dimensionality control, and evaluation design. The workflow applies iterative recursive feature elimination combined with ensemble training to identify predictive features while mitigating overfitting. Features exhibiting strong phylogenetic linkage or restricted strain distribution are systematically filtered, explicitly addressing the risk that models learn lineage signatures rather than interaction determinants. Across extensive parameter sweeps, feature-selection strategy and training protocol were found to exert a larger influence on performance than the precise choice of genomic representation, underscoring that workflow design, rather than architectural complexity, dominates model behaviour under sparse supervision.

Prediction is performed using ensemble ML classifiers, with CatBoost gradient-boosted decision trees [89] selected as the final architecture based on stability and performance across datasets [69]. Interestingly, class imbalance is addressed through phage-specific inverse-frequency weighting, reflecting a deliberate decision to avoid reshaping class geometry in ways that may inflate apparent performance. Importantly, train-test splits are constructed using hierarchical clustering of host strains, ensuring that closely related strains are not trivially shared across folds. PAML explicitly evaluates multiple generalization regimes, including prediction for unseen strains, unseen phages, and entirely unseen strain–phage pairs, providing a stringent assessment of real-world deployment scenarios than random pairwise splitting.

Evaluation strategy is central to the PAML framework. The authors prioritize Matthews correlation coefficient (MCC) and precision-recall (PR) analysis, while explicitly cautioning against reliance on AUROC alone in sparse interaction matrices. Performance scales strongly with dataset size and interaction coverage, with the largest gains observed when datasets are expanded within genera rather than merged indiscriminately across distant taxa [69]. This scaling behaviour reinforces a core design premise of PAML: in the absence of strong mechanistic priors, data breadth, diversity, and outcome consistency become the dominant determinants of strain-level predictive power.

Despite its agnostic design, PAML systematically interrogates biological relevance using feature-attribution analyses based on SHapely Additive exPlanations [90]. Predictive signal maps back to protein families and k-mers corresponding to known receptors, defence systems, and anti-defence mechanisms, supported by experimental validation using large-scale host-range assays and transposon sequencing screens [69]. Together, these results demonstrate that biologically meaningful determinants can be recovered without explicit mechanistic priors when evaluation and generalization are handled conservatively.

Taken together, DeepPBI-KG and PAML illustrate two distinct pathways toward adsorption-agnostic strain-level PHI prediction. DeepPBI-KG expands the hypothesis space through engineered genomic features within a species-specific context, while PAML distributes predictive signal across genome-wide representations under conservative evaluation and generalization regimes. Both demonstrate that relaxing explicit mechanistic assumptions can recover strain-level signal, but also reinforce that predictive performance remains tightly coupled to dataset coverage, outcome structure, and workflow design rather than architectural expressiveness alone.

### Phage–bacterium interaction predictor: an end-to-end deep learning framework

PBIP, introduced by Ma *et al.* [27], represents the most fully representation-driven strain-level PHI predictor reported to date. In contrast to feature-engineered or hybrid approaches, PBIP seeks to infer phage-host compatibility directly from pretrained protein sequence embeddings, without explicit specification of receptors, RBPs, or defence systems.

PBIP was developed using a tightly controlled, strain-resolved PHI dataset comprising 120 *K. pneumoniae* clinical isolates tested against 104 *Klebsiella* phages. Initial spot-test screening was followed by planktonic killing assays to confirm bactericidal activity, yielding a dense and internally consistent PHI matrix comprising 12 480 phage–host pairs [27].

At the representation level, PBIP encodes protein sequences from both phages and bacterial hosts using the pretrained pLM UniRep [91]. Rather than selecting or annotating specific infection determinants, PBIP applies a two-stage aggregation strategy: UniRep embeddings are first computed for each predicted protein and then mean-pooled across all proteins within a phage and within a host, respectively. These organism-level representations are subsequently concatenated to form a composite phage-host input vector for classification. The concatenated embeddings are processed by a deep neural network comprising a convolutional module for local feature extraction, a bidirectional gated recurrent unit to model longer-range dependencies, and an attention mechanism to weight salient features prior to final prediction [27].

This design introduces several strong but explicit inductive assumptions. It assumes that pretrained protein embeddings encode information relevant to infection compatibility despite being optimized for general sequence modelling rather than PHI-specific tasks, and that aggregation across all proteins yields a meaningful summary of phage-host compatibility despite large disparities in proteome size between bacterial hosts and phages. PBIP does not explicitly normalize for proteome size or constrain which proteins contribute most strongly to predictions, leaving the biological attribution of learned signal unresolved. While this abstraction enables end-to-end learning without manual feature engineering, it also compresses interaction structure in ways that complicate mechanistic interpretation.

A defining feature of PBIP is its explicit treatment of extreme class imbalance. Positive interactions are augmented using the synthetic minority oversampling technique (SMOTE) [92] directly within the learned embedding space. Ablation analyses show that removal of this augmentation step results in a substantial reduction in predictive performance (~7%–8%) [27], underscoring both the effectiveness of oversampling and the sensitivity of DL architectures to class balance under sparse positive sampling [93]. Notably, similar sensitivity to oversampling was observed when benchmarking against retrained species- and genus-level predictors, suggesting that part of PBIP’s apparent gains reflect reshaped decision boundaries under sparse supervision rather than improved biological resolution *per se*.

Across benchmarking experiments, PBIP outperformed several existing PHI predictors on strain-level tasks, particularly when training and test data shared high sequence similarity [27]. Performance declined as sequence divergence increased, indicating reduced robustness when extrapolating beyond densely sampled, system-specific interaction spaces.

Taken together, PBIP illustrates both the promise and the current constraints of fully end-to-end DL for strain-level PHI prediction. Under tightly controlled conditions with dense, high-quality training data, representation-driven architectures can achieve strong predictive performance without explicit mechanistic feature engineering. However, sensitivity to dataset composition, reliance on synthetic augmentation, and limited biological attribution indicate that architectural flexibility alone does not resolve the fundamental challenges of strain-level PHI modelling.

## Bridging practical and conceptual gaps in strain-level PHI prediction

Recent advances in strain-level PHI prediction demonstrate that meaningful computational signal can be recovered when modelling strategies are aligned with biological context, assay design, and data availability. As reviewed across Section 2, several approaches achieve encouraging strain-resolved performance under carefully controlled experimental conditions. However, these successes are largely confined to narrow experimental and taxonomic settings, revealing structural constraints that persist across modelling paradigms, independent of architectural complexity.

Progress towards precise, interpretable, and generalizable strain-level PHI prediction therefore requires reframing current limitations in terms of downstream application requirements rather than individual model designs. Clinical phage therapy, in particular, represents a high-value but high-complexity use case, demanding rapid and reliable phage prioritization across diverse, often under-characterized bacterial strains. While regulatory and logistical considerations associated with clinical translation lie beyond the scope of this review (but are discussed elsewhere [94–96]), substantial modelling challenges remain intrinsic to strain-level PHI prediction itself; particularly with respect to data availability, infection complexity, evaluation at strain resolution, and generalizability across experimental and biological contexts.

### Data and label constraints

At strain resolution, the primary constraint on PHI prediction is not model architecture but the structure, density, and biological resolution of available outcome data. Discriminating between closely related bacterial strains requires PHI matrices that are dense, internally consistent, and phenotypically informative. In practice, such datasets remain rare. Even intensively curated experimental screens typically yield only a small number of confirmed productive infections embedded within a vast space of non-infective, ambiguous, or untested outcomes, reflecting both the biological reality of narrow host ranges and the limitations of scalable infection assays [6, 40, 67].

This data landscape imposes two interrelated challenges. First, strain-level PHI labels are intrinsically uncertain: many phage–host pairs are never assayed, others yield assay-dependent or context-specific outcomes, and negative results may reflect experimental conditions rather than true biological incompatibility. Second, this uncertainty manifests statistically as extreme and structured class imbalance, with positive interactions forming a sparse minority unevenly distributed across phages, hosts, and lineages. Importantly, this imbalance is not a technical artefact, but a direct consequence of how strain-level PHI data are generated, labelled, and interpreted [97].

A common modelling response to sparcity is the binarization of semi-quantitative infection outcomes into permissive and non-permissive classes. For example, PxPC [6] and subsequently PAML [69] treat any non-zero MLC score as productive infection, increasing the number of positive PHIs available for training and stabilizing optimization under extreme imbalance. However, this strategy necessarily collapses heterogeneous biological states (including partial lysis, inefficient infection, and abortive processes such as lysis from without) into a single positive label. Similar ambiguities arise in spot assays, where treatment of partial or turbid plaques can materially affect class assignment. While such simplifications facilitate model training and evaluation, they discard information about infection efficiency, intracellular progression, and context dependence.

Different modelling frameworks operationalize these ambiguities in different ways. Feature-based and hybrid models such as PxPC and PhageHostLearn primarily address imbalance at the optimization and evaluation stages through class weighting, stratified sampling, and threshold calibration [6, 67]. More data-driven approaches extend imbalance correction into the data domain itself, as illustrated by DeepPBI-KG’s inferred negatives [68] and PBIP’s use of SMOTE within embedding space [27]. The strong sensitivity of performance to these choices, observed both for strain-level models and retrained higher-level predictors [18, 19, 27], underscores that imbalance mitigation can substantially reshape decision boundaries without necessarily improving biological resolution.

PAML adopts a more conservative stance, treating imbalance as an inseparable consequence of incomplete outcome knowledge [69]. By prioritizing restrained sampling, careful outcome definition, and imbalance-aware evaluation metrics such as PR analysis and MCC, it reduces susceptibility to skew-driven artefacts, implicitly accepting that predictive power scales with dataset breadth, diversity, and coverage rather than architectural expressiveness alone.

Representation choice is tightly coupled to these data constraints. Feature-informed approaches deliberately restrict hypothesis space to biologically interpretable determinants of infection, enabling tractable learning under sparse and noisy supervision. In contrast, feature-agnostic and relational models developed at broader taxonomic scales, including graph-based approaches such as PHIS-GAE and MI-RGC [22, 98], rely on dense interaction graphs or extensive local sequence relationships to distribute learning signals. These assumptions degrade at strain resolution precisely because PHI matrices are sparse, uneven, and often dominated by a small number of highly connected nodes, increasing the risk that models amplify degree- or connectivity-driven sampling artefacts rather than resolve true strain-specific compatibility.

Large-scale genomic foundation models, such as Evo and its successor Evo 2 [99, 100], introduce a distinct representational paradigm by learning broad sequence distributions rather than task-aligned infection determinants. By encoding genome-wide evolutionary constraints without requiring explicit annotation, these models capture a comprehensive view of known genomic variation across all domains of life. However, whole-genome representations also encompass extensive sequence variation unrelated to infection compatibility, which may dilute relevant signal under the sparse and highly imbalanced supervision of host-range datasets. While recent work demonstrates that foundation models can generate biologically viable synthetic genomes [101], genome viability is not coupled with strain-resolved infection outcomes. A critical next step will therefore be to evaluate Evo-based models fine-tuned on all available curated phage host-range data, to assess whether their global genomic knowledge can be effectively aligned with interaction-specific supervision. At present, foundation models are complementary to, rather than replacements for, outcome-driven PHI predictors.

Taken together, these considerations define an upper bound on what strain-level PHI models can learn from existing datasets. Even with optimal representations and careful evaluation, predictive performance remains constrained by outcome ambiguity, imbalance, and incomplete sampling. These limitations motivate a second, orthogonal challenge: whether current modelling strategies meaningfully capture the biological processes that determine infection at strain resolution.

### Infection biology beyond adsorption

Addressing data and label constraints is necessary for strain-level PHI prediction, but it is not sufficient. These factors define what models can learn, not whether their predictions faithfully reflect infection biology. Productive phage infection is a multistage, context-dependent process shaped by numerous host and phage factors that vary at strain resolution and are sensitive to environmental conditions. Growth phase, host physiology, and experimental context can modulate receptor accessibility, defence activity, and intracellular pathways, leading to assay-dependent or discordant outcomes even among closely related strains [102–105]. As a result, strain-level PHI labels often represent a coarse projection of a deeper, multi-layered infection trajectory that may not generalize across assays or translate *in vivo*.

In principle, training datasets that capture this complexity through quantitative, time-resolved, or assay-consensus outcomes would provide a more faithful substrate for modelling. In practice, the experimental burden required to generate such data at scale remains prohibitive, as each added layer of biological resolution imposes a disproportionate cost in throughput and standardization. This tension motivates a central question for strain-level PHI prediction: how much biological detail is required to generate predictions that are clinically useful, rather than biologically exhaustive?

Most existing strain-level models respond to this trade-off through explicitly reductive strategies. PxPC and PhageHostLearn prioritize adsorption determinants because these features were empirically shown to be among the most predictive and reproducible signals in their respective systems [6, 67]. By focusing on well-characterized receptor-associated features, these frameworks enable tractable learning under sparse supervision, but necessarily under-represent downstream intracellular barriers to infection [106]. DeepPBI-KG and PBIP relax reliance on curated adsorption features through engineered genomic descriptors and end-to-end embeddings, respectively, yet remain constrained by the coarse biological resolution of the underlying outcome labels [27, 68].

An important and often overlooked consequence of these reductive strategies lies in how protein-level signal is aggregated prior to prediction. In PhageHostLearn, information from multiple RBPs or capsule-associated proteins is compressed into fixed-length vectors via pooling and concatenation [67]. While this stabilizes learning under sparse supervision, it collapses heterogeneity among individual proteins, discarding information about dominance, combinatorial effects, or context-dependent contributions. As a result, such models learn average compatibility between phage and host adsorption machinery rather than resolving protein-specific determinants that may drive strain-level susceptibility.

This limitation becomes more pronounced as models rely increasingly on representation learning. PBIP demonstrates that pretrained pLM embeddings can recover strain-level signals when paired with dense, system-specific datasets [27]. However, sequence-only representations encode infection determinants indirectly, abstracting away explicit biophysical properties of RBP-receptor recognition such as conformational flexibility, binding interfaces, and surface electrostatics, which are known to influence host specificity at fine scales [107–110]. Consistent with this, PHPRBP shows progressively stronger performance as prediction is relaxed from strain to higher taxonomic ranks, suggesting that rich sequence representations amplify adsorption-driven signal at coarse scales while failing to resolve fine-grained strain-level determinants [26].

Broader analyses of pLM scaling reinforce these limitations. Mutation-effect prediction performance is not monotonic with model size, peaking at intermediate perplexity and degrading at both extremes, where residue-level discrimination collapses [111]. Notably, viral proteins show systematically lower performance even at comparable perplexity levels, consistent with distribution shift and distinct evolutionary pressures. Together, these findings caution against assuming that pLM embeddings inherently capture strain-level, infection-relevant variation, particularly for phage RBPs.

Structure-aware approaches provide a complementary avenue to probe these limits. PHIStruct [112] integrates predicted three-dimensional protein structure with sequence information using SaProt embeddings [113–115], making aspects of molecular recognition more explicit than sequence-only representations. Although currently evaluated at genus-level resolution, it illustrates how structural context may recover binding-specific information that sequence embeddings capture only indirectly. However, uncertainty in predicted structures, task misalignment, and receptor diversity constrain universal applicability. In systems dominated by non-proteinaceous or structurally heterogeneous receptors, such as LPS-mediated adsorption in *Escherichia* and *Pseudomonas*, direct receptor modelling remains challenging. PxPC nonetheless demonstrates that carefully curated adsorption features can still provide strong predictive signal in such systems [6], suggesting that structural refinement is best viewed as augmentative rather than substitutive.

Alternative frameworks respond to infection complexity by deliberately relaxing mechanistic assumptions rather than attempting to resolve specific stages of infection. PAML [69] exemplifies this epistemically conservative approach. By explicitly acknowledging outcome ambiguity and constraining predictive claims through careful outcome definition, restrained handling of negatives, and imbalance-aware evaluation, PAML recognizes that inferring fine-grained infection stages from incomplete strain-level PHI matrices risks overinterpretation rather than improved biological insight.

The value of this restraint is particularly evident in biologically complex hosts such as *Pseudomonas aeruginosa*. PAML reports its lowest strain-level predictive performance in this genus [69], likely reflecting both smaller, more heterogeneous datasets and the intrinsically multifactorial infection biology of *P. aeruginosa*, in which determinants are genomically dispersed, variably expressed, and often poorly annotated [30, 116, 117]. Importantly, this relatively reduced performance does not indicate model failure; rather, it exposes the intrinsic difficulty of resolving strain-level compatibility under such conditions.

Finally, PHIs unfold within a dynamic coevolutionary landscape that varies across temporal and spatial scales [118]. Current strain-level models are trained on static datasets and therefore fail to capture evolutionary turnover in receptors, defence systems, and phage counter-adaptations [119–121]. While some bacterial populations exhibit spatiotemporal stability over multi-year periods [122], it remains unclear how generalizable this stability is across species and ecological contexts. This uncertainty complicates decisions about model update frequency and clinical durability. Iterative, feedback-driven updating strategies such as periodic re-training or re-ranking using contemporary data, may partially mitigate this limitation, but impose substantial experimental and computational costs. Ultimately, richer longitudinal PHI datasets will be required to fully integrate evolutionary dynamics into strain-level prediction frameworks.

### Evaluation, benchmarking, and apparent strain-level performance

As strain-level PHI prediction models grow in architectural sophistication, evaluation methodology becomes a central determinant of perceived success. Apparent gains in predictive accuracy do not necessarily reflect genuine resolution of fine-scale host specificity, particularly in sparse, imbalanced, and system-specific PHI datasets. Under these conditions, models can achieve strong headline metrics by exploiting statistical regularities that are orthogonal to the biological question of strain-level compatibility, creating an illusion of strain-level performance driven by dataset structure rather than mechanistic inference.

A core challenge is that many commonly reported performance metrics were not designed for strain-resolved inference. Metrics such as accuracy and AUROC remain informative under balanced or moderately imbalanced conditions, but they are poorly aligned with strain-level PHI matrices, where positive interactions are rare and unevenly distributed (Section 3.1). In such settings, AUROC can remain deceptively high even when precision for the minority class is low, as it disproportionately rewards true-negative discrimination and is insensitive to false-positive burden. As a result, models that perform poorly in practical screening scenarios may nonetheless appear competitive when evaluated using AUROC alone [123].

Metrics derived from PR analysis provide a more informative perspective in rare-positive regimes, as they directly capture the trade-off between sensitivity and false discovery [123]. Similarly, the MCC offers a single-value summary that incorporates all four confusion-matrix terms and is less dominated by the majority class. Reflecting this, PAML explicitly prioritizes PR metrics and MCC over AUROC [69], while PxPC, DeepPBI-KG, and PBIP also emphasize PR-based evaluation [6, 27, 68]. Together, these choices reflect a growing consensus that imbalance-aware metrics are essential for meaningful assessment of strain-level PHI prediction performance.

Metric choice alone, however, is insufficient to ensure valid evaluation. A more subtle but equally consequential source of inflated performance arises from how training and test splits are constructed. Random pairwise splitting of PHI matrices can place highly similar bacterial strains or closely related phages on both sides of the train-test boundary, particularly in datasets derived from single laboratories or narrowly sampled cohorts. Under such conditions, models may achieve high test-set performance by learning lineage-level or system-specific signatures rather than determinants that truly distinguish permissive from non-permissive strains.

Several studies have begun to address this risk explicitly. For example, PAML quantified genomic similarity between phages in its training and test sets using Dashing [124], reporting moderate overlap and concluding that the resulting split was appropriate for evaluation [69]. While such analyses represent an important step toward transparency, the absence of established similarity thresholds and the continuous nature of genomic relatedness complicate interpretation. Without explicit controls on phylogenetic or genomic similarity, strain-level evaluation risks collapsing into a softened form of species- or lineage-level prediction. This limitation applies across modelling paradigms, affecting feature-based, hybrid, and DL approaches alike.

The construction and treatment of negative examples introduce an additional layer of complexity. As discussed in Section 3.1, strain-level PHI datasets contain a mixture of verified negatives, untested pairs, and context-dependent outcomes. Different studies operationalize this ambiguity in different ways, ranging from conservative masking to aggressive negative inference or synthetic resampling. Analyses in DeepPBI-KG and PBIP illustrate that imbalance mitigation strategies can substantially reshape decision boundaries and metric values, not only for the proposed models but also for broader taxonomic baselines [27, 68]. Improved performance under such regimes may therefore reflect altered class geometry rather than enhanced biological resolution.

Together with split design and metric choice, these considerations suggest that evidence for strain-level predictive capability should depend on the generalization regime being claimed, not solely on evaluation within a strain-resolved matrix. At minimum, evaluation should use grouped splits that limit leakage of closely related strains (and, for global models, closely related phages), rather than random pairwise splits. Stress tests such as leave-one-host-lineage and (where applicable) leave-one-phage-lineage validation better probe robustness to novelty. Where comparable datasets exist, cross-dataset or cross-laboratory evaluation provides the strongest test of clinical transfer, but should be interpreted explicitly as domain shift, given assay and label heterogeneity.

Within this context, benchmarking strain-level models against broader taxonomic predictors can be informative, but only if interpreted cautiously. PBIP’s comparison against species- and genus-level models such as PredPHI and PHIAF illustrates this point: competitive performance under certain evaluation regimes indicates that higher-level models can recover predictive signal in strain-resolved datasets, but it does not imply resolution of strain-specific determinants [27]. Rather, such comparisons establish practical baselines under shared evaluation constraints and underscore the difficulty of disentangling true strain-level discrimination from implicit exploitation of phylogenetic structure or dataset composition.

### Towards generalizable and clinically useful models

The preceding sections highlight a central tension in strain-level PHI prediction: models can achieve strong apparent performance within narrowly defined experimental settings [6, 27, 67–69], yet fail to generalize to the heterogeneous and uncertain conditions encountered in clinical practice. As discussed, this limitation is structural rather than architectural, arising from sparse outcome data, assay-dependent labels, and evaluation regimes that can overestimate true strain-level resolution. Generalizability in this context should therefore be understood not as universal predictive accuracy, but as the capacity to provide robust, uncertainty-aware, and interpretable decision support when confronted with novel strains, incomplete data, and shifting biological conditions.

This distinction is particularly salient for clinical phage therapy, where model outputs may directly influence experimental prioritization or treatment decisions. Predictive frameworks must extrapolate beyond their training distributions while remaining transparent and interrogatable to support user trust and regulatory expectations [125–129]. Furthermore, patient-derived bacterial isolates may differ substantially from reference strains, carrying novel resistance determinants, surface structures, or regulatory features absent from model training datasets [130–133]. Likewise, candidate therapeutic phages may exhibit infection strategies not captured in the initial assay design or belong to genera that are underrepresented in the original model training datasets [134]. Under such distributional shift, even models with strong internal validation may degrade sharply, making overconfident reliance on static *in silico* predictions a clinical risk.

As outlined in Section 3.2, improving generalizability will likely require closer engagement with biological and experimental complexity. Training data must offer not only scale, but relevance: binary outcomes derived from spot tests or single-timepoint assays capture only a limited slice of the infection process and often correlate poorly with therapeutic efficacy [52]. In clinical workflows, actionable PHI labels typically reflect consensus across multiple assays, including spot tests, time-kill curves, and growth inhibition across phage-to-bacteria ratios [47]. Where generating such datasets at scale is impractical, the feasibility of fully autonomous strain-level PHI prediction remains uncertain.

A pragmatic alternative is to embed PHI models within hybrid, feedback-driven workflows. In this design, *in silico* predictions guide initial phage prioritization, followed by rapid phenotypic screening on the target isolate, with empirical outcomes used to recalibrate or re-rank candidates. Such hybrid systems align naturally with clinical phage therapy practice, repositioning strain-level PHI models as adaptive decision-support tools rather than standalone arbiters of efficacy: a framing, i.e. more consistent with current data realities and biological uncertainty.

Key Points

- Strain-level PHI prediction is feasible under controlled conditions, particularly in well-characterized phage–host systems, but apparent performance is highly sensitive to dataset composition, evaluation regime, and experimental context, limiting generalizability across taxa, laboratories, and clinical settings.
- The dominant constraint is structural rather than architectural: strain-level PHI datasets are sparse, imbalanced, and assay-dependent, and predictive performance is tightly coupled to outcome definition, label resolution, and negative handling rather than model complexity alone.
- Infection outcomes collapse multistage, context-dependent biology, and increasing representational flexibility does not inherently resolve this limitation. Structure-aware descriptors and annotation-light gene–genome compatibility can enhance prediction in selected systems, but no single representation universally captures strain-level specificity.
- Evaluation choices critically shape perceived strain-level performance. Conventional metrics and random test-train splits can overestimate resolution by exploiting phylogenetic structure, sampling density, or class imbalance.
- Clinically useful strain-level PHI prediction is best framed as decision support, not automation. Hybrid, modular systems that combine mechanistic priors, learned representations, and iterative phenotypic feedback offer the most credible path towards interpretable, adaptable, and translationally relevant models.

## Data Availability

No new data were generated or analysed in support of this research.
